# Managing Surgical Risks in Hemophilic Elbow Arthropathy: An In-Depth Case Study and Literature Review

**DOI:** 10.3390/healthcare12171776

**Published:** 2024-09-05

**Authors:** Gianluigi Pasta, Salvatore Annunziata, Roberta Ruggieri, Dario Abruzzi, Paolo Arrigoni, Eugenio Jannelli, Francesco Benazzo, Luisella Pedrotti, Erika Maria Viola, Emérito Carlos Rodriguez-Merchan, Mario Mosconi

**Affiliations:** 1 Orthopedics and Traumatology Clinic, IRCCS Policlinico San Matteo Foundation, 27100 Pavia, Italy; 2 Sezione di Chirurgia Protesica ad Indirizzo Robotico-Unità di Traumatologia dello Sport, U.O. Ortopedia e Traumatologia Fondazione Poliambulanza, 25124 Brescia, Italy; 3 Department of Clinical, Surgical, Diagnostic and Pediatric Sciences, University of Pavia, 27100 Pavia, Italy; 4 Centre for Health Technologies, University of Pavia, 27100 Pavia, Italy; 5 Department of Orthopedic Surgery, La Paz University Hospital-IdiPaz, 28046 Madrid, Spain

**Keywords:** hemophilia, elbow arthropathy, surgical management, surgical complications

## Abstract

This study presents a detailed case analysis of a 40-year-old male patient with hemophilia A and severe chronic elbow arthropathy, exploring the surgical challenges and outcomes within the context of the current literature. The patient, with a history of multiple comorbidities including Hodgkin’s lymphoma and cardiomyopathy, exhibited significant joint damage and functional impairment. A comprehensive approach was employed, collecting all relevant clinical data, including radiographic and MRI findings, to inform treatment decisions. Clinical findings and treatment decisions are presented as they occurred in real time, simulating the clinical reasoning process. Subsequent references to the clinical and instrumental findings as well therapeutic interventions are discussed in light of the current literature to reinforce the decision-making framework. This report underscores the importance of multidisciplinary care in optimizing patient outcomes and contributes to the ongoing discourse on the management of advanced musculoskeletal conditions in hemophilic patients. The findings emphasize the necessity for early intervention and specialized care to mitigate complications and improve long-term prognosis.

## 1. Introduction

Hemophilia, an X-linked recessive coagulopathy affecting males globally, is classified as Hemophilia A (factor VIII deficiency, 1:5000) and Hemophilia B (factor IX deficiency, 1:20,000), with severity determined by functional clotting factor levels. Hemophilic arthropathy (HA) is the leading cause of morbidity in severe hemophilia patients with factor VIII or IX levels under 0.01 UI mL^−1^ [1]. The average age for the first hemarthrosis episode is between 17 months and 2.2 years [2]. By age 25, 90% of severe hemophilia patients exhibit chronic degenerative changes in 1–6 joints [3], with the knee (50.9%), ankle (42.8%), elbow (38.5%), and shoulder (13.3%) being most affected.

Elbow arthropathy impacts 13% to 87% of hemophilia patients [4], ranking second or third in prevalence depending on the study [5]. 

Hemophilic elbow arthropathy typically presents with a range of clinical features that significantly impact the patients’ quality of life. These include reduced range of motion, pain, muscle atrophy, and strength loss in the affected elbow, alongside axial changes that complicate daily activities and functional capacity [6].

Hemarthrosis, often the initial manifestation of hemophilia, is anatomopathologically characterized by chronic synovitis and cartilaginous damage [7]. Acute intra-articular bleeding stretches the capsule, causing synovial reaction and inflammatory cell infiltration [8]. Recurrent bleeding reduces blood resorption capacity, leading to intra-articular hemosiderin accumulation [7], which induces local expression of the proto-oncogene c-myc in synovial cells, activating proteins like VEGF, MDM2, MMP9, and IL1. This causes mechanical damage, local tissue damage, chondrocyte apoptosis, and reduced proteoglycan synthesis in degenerative joints [9,10,11,12,13,14]. Evidence suggests that even a single intra-articular bleeding episode causes long-term damage to chondrocytes and matrix, with the direct blood effect on cartilage preceding the indirect effect of synovial inflammation [15,16,17]. These processes lead to persistent hemophilic synovitis, triggering a cycle of hemarthrosis and synovitis, which, if unbroken, causes chondrocyte death, joint cartilage destruction, and HA within a few years, significantly impacting quality of life, especially in severe hemophilia cases and those with inhibitor antibodies against the infused deficient factor [18]. 

Recurrent bone fracture site bleeding or sub-periosteal hemorrhage without effective factor replacement may lead to hemophilic pseudotumors [19]. A small subset, approximately 1–2%, of hemophiliac patients, predominantly those with recurrent soft-tissue hemorrhages, develop hemophilic pseudotumors. Inadequate blood product reabsorption results in clotted blood surrounded by fibrous tissue, presenting as a mass effect. Patients often report chronic deep pain alleviated by rest, with vascular or neurological mass effect symptoms, especially limb paresthesia. Superficial masses may cause pain during movement and joint impairment [20]. The patient exhibited typical elbow arthropathy symptoms, including pain, stiffness, and local swelling.

The primary approach for managing arthritic elbow conditions involves several key elements, including rest, the use of nonsteroidal anti-inflammatory drugs, activity modification, and preventing the progression of the disease. However, modifying activities can be challenging for individuals engaged in manual labor or athletic pursuits. In addition to these measures, physical therapy and the use of dynamic hinged or static progressive splinting are also typically recommended.

Viscosupplementation is a treatment option for arthritic elbow conditions that affects the main body joints. When nonsurgical treatments are unsuccessful, surgical intervention may be necessary, taking into account factors such as the patient’s age, functional demands, the cause of the arthritis, and the severity of the condition. In cases of moderate degenerative changes, arthroscopic or open synovectomy (the surgical removal of the synovial membrane of a joint) may be recommended. Severe elbow arthritis may require more extensive procedures, such as distraction interposition arthroplasty or total elbow arthroplasty (TEA), a joint replacement surgery for the elbow, as described in the relevant literature [21]. 

This case report follows a unique structure designed to engage the reader by presenting clinical events, imaging findings, and therapeutic decisions as they occurred in real time. By integrating these real-time decisions with references to the existing literature, the reader is invited to reflect actively on the management options and their outcomes, thus enhancing the educational value of the case.

To support and contextualize these clinical decisions, we conducted a comprehensive narrative review. A thorough search of the literature was carried out using the MEDLINE and Embase databases to identify studies related to hemophilic elbow arthropathy. Studies were selected based on their level of evidence, with preference given to those of higher quality. The review focused on the outcomes of conservative and surgical treatments, patient-reported outcomes, complications, and the conversion to stabilization or revision surgery, thereby reinforcing the evidence-based approach of our clinical decision-making.

The decision to publish the patient’s clinical course was made retrospectively, after all treatments had been completed. Consequently, obtaining prior ethical approval was not feasible. Moreover, the patient has provided explicit consent for the use of their sensitive data for scientific purposes.

## 2. Case Presentation

This case involves a 40-year-old Caucasian male with severe hemophilia A, who presents with elbow arthropathy complicated by multiple comorbidities and a history of smoking. The patient underwent factor replacement prophylaxis for elbow arthropathy and was employed in an office setting. The patient exhibited a restricted range of motion in the right elbow, with flexion limited to 100 degrees and extension to 20 degrees, affecting daily activities, but did not engage in contact sports during leisure time.

### 2.1. History 

**Figure 1:**
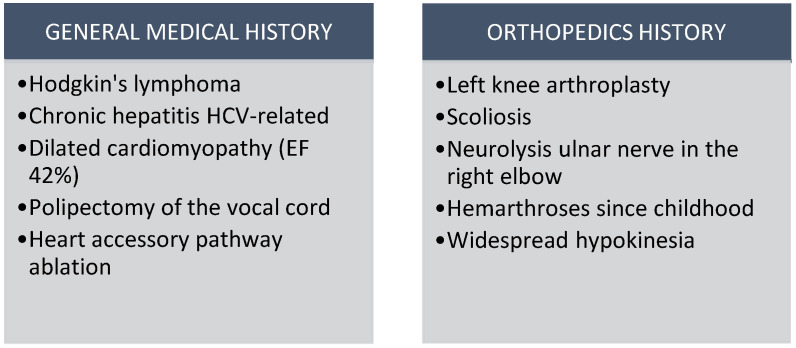
Patient’s medical history. (HCV: Hepatitis C virus) (EF: Ejection Fraction).

The patient in question had a medical history that included not only hemophilia, but also Hodgkin’s lymphoma that was treated with the ABVD protocol (utilizing doxorubicin, bleomycin, vinblastine, and dacarbazine). Additionally, the patient had chronic hepatitis C, dilated cardiomyopathy with an ejection fraction of 42%, and widespread hypokinesia, as well as moderate mitral insufficiency with left atrial dilatation. The patient had also undergone a previous polypectomy of the vocal cord, occult right posterior-septal accessory pathway ablation with radio frequency (RF), scoliosis, and left knee arthroplasty for a HA with varus deformity. The patient additionally received conservative treatments, undergone neurolysis of the ulnar nerve on the right elbow at another hospital, and experienced hemarthroses since childhood (as depicted in Figure 1).

Comorbidities have a significant impact on both the clinical presentation and surgical outcomes for patients with hemophilia, as demonstrated by Utukuri et al. [22]. In particular, a history of lymphoma and recurrence of this cancer during follow-up can complicate the situation further.

### 2.2. Clinical Presentation

In March 2013, the patient sought treatment at our Orthopedic and Trauma Surgery department, complaining of continuous pain (Visual Analog Scale [VAS] score of 6), swelling, and limited active range of motion in the right elbow. The patient’s range of motion was 20–100 degrees for flexion–extension and 0–45 degrees for prono-supination, which impaired his daily activities. Fortunately, the patient did not have any problems with other joints, including his left total knee arthroplasty (TKA). The patient’s right shoulder had a normal range of motion without any limitations or pain.

Evidence from the Scientific Literature

Assessing the involvement of other joints is crucial, as it impacts overall functional outcomes. Malhotra’s study indicated that most patients with elbow arthropathy also had knee joint involvement, likely due to the repetitive load on the elbow from using walking aids. In their study, about 25% of patients with severe elbow arthropathy had other joint involvement. Aronstam concluded that patients experiencing pain, tenderness, and a loss of over 50% of ROM have a poor long-term prognosis. Gamble found that older hemophilia patients (>25 years) had significantly greater motion loss in the elbow and wrist than younger patients, emphasizing early treatment’s importance [22]. About 80–90% of hemophilia patients without primary prophylaxis develop elbow arthropathy because the upper limbs assist in ambulation during lower-limb bleeding episodes. This assistance includes transitioning from sitting to standing and using walking aids such as sticks, crutches, or walkers [6]. Although the elbow is not a weight-bearing joint, early ROM limitations rarely affect daily activities [23,24]. As joint deterioration progresses, the humerus-ulnar joint is impacted, restricting flexion and extension, thus affecting daily activities. Bone deformities in some cases may lead to ulnar nerve neuropathy [25]. 

### 2.3. Further Investigations

**Figure 2:**
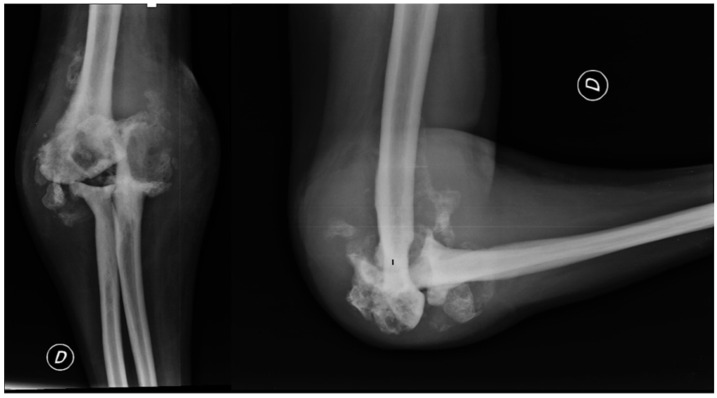
X-ray anteroposterior and lateral views of the right elbow. First radiological presentation with subversion of the local anatomy of the bones and soft tissue around the elbow joint.

The patient’s clinical presentation necessitated a right elbow radiograph, which revealed substantial morphological changes in the joint’s bone structures, including osteolytic areas in the bone, as shown in Figure 2. The radiograph also displayed calcification of the periarticular soft tissue and radial head dislocation. Furthermore, the patient experienced an enlarging ulnar elbow mass, which was attributed to recurrent intra-articular bleeding affecting the periarticular soft tissue.

**Figure 3:**
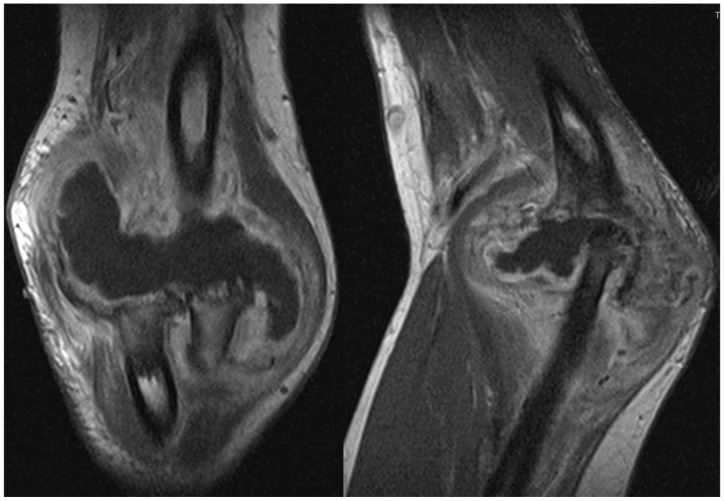
Magnetic resonance imaging (MRI) coronal and sagittal T1 weight views of the right elbow. Involvement of periarticular soft tissue with a large mass through the articulation.

The patient also underwent magnetic resonance imaging (MRI) with and without contrast medium, which demonstrated subluxation between the articular surfaces of the elbow joint, edema and contrast enhancement in the spongy bone of the distal third of the humerus and proximal third of the radius, and a periosteal reaction at the humeral site without identifiable bone lesions, as shown in Figure 3. Additionally, the imaging revealed an abundant layer of joint effusion, with blood clots and loose bodies, at the joint level. The synovium appeared thickened and full of contrast enhancement, and the periarticular soft tissues also displayed thickening with edema and soft contrasting infiltration on the proximal ulnar side. In light of the known basic arthropathy and the patient’s clinical presentation, these radiological findings suggested an arthritic arthro-synovitis picture with soft tissue involvement, without excluding an infectious nature, rather than osteomyelitis, as there was no cavity of necrotic bone with fistula and bone marrow inflammation.

Evidence from the Scientific Literature

Conventional radiography, ultrasound, and MRI are the primary imaging modalities for examining HA. The Pettersson score and Arnold Hilgartner scale are widely used classifications for HA via radiography [26,27]. These classifications rely on plain films to identify osteoporosis, bone cysts, osteonecrosis, bone fusion, joint space irregularities and narrowing, and epiphyseal overgrowth. However, these methods are limited in evaluating soft tissues.

Plain films are useful for assessing late arthropathy but may not detect early changes [28]. The correlation between clinical and radiographic features remains unclear. Ultrasound can precisely visualize soft tissues and guide infiltrations [29]. Elbow synovitis appears as a hypoechogenic line that thickens and becomes irregular with inflammation, showing increased vascularity on color or power Doppler. In normal joints, the synovial membrane is barely visible on ultrasound but becomes distinct in inflammation. Variability in ultrasound interpretation poses challenges for standardization and quantification; nonetheless, it is an effective and economical tool for monitoring hemophiliac joints, with increasing usage.

MRI is considered the most sensitive test for arthropathy and remains more sensitive than other imaging modalities, despite being less sensitive in the elbow compared to other joints. Evaluations should include coronal, sagittal, and axial examinations. Cross et al. recommend a T1 sequence for osteochondral lesions, with unenhanced gradient echo best for viewing synovium, cartilage, and hemosiderin deposition. T2 gradient echo is effective for identifying acute and chronic bleeding and distinguishing between simple effusion and hemorrhage [28]. 

MRI can identify even minor changes in the affected joint, significantly impacting patient management [30]. Several MRI-based rating scales for HA have been suggested [31,32,33,34]. The revised 2005 International Prophylaxis Study Group (IPSG) scale, based on the IPSG MRI scale, uses a single scoring system to include soft tissue changes like effusion and hemarthrosis, distinguishing between soft tissue and osteochondral changes to better reflect disease progression [28]. Computed tomography (CT) focuses on the primary structures and modifications of bones [35]. Both CT and MRI can determine the extent of hemophilic pseudotumors, which are chronic, encapsulated hemorrhagic fluid collections that typically destroy bone and can grow significantly [30]. Three-dimensional (3D) CT aids in surgical planning for total elbow arthroplasty (TEA), helping estimate implant size and placement [36]. Angiography with arterial embolization, despite being invasive and carrying some risk, reveals vascular blush, false aneurysms, true aneurysms, and arteriovenous shunts causing bleeding. Although spontaneous periarticular aneurysms causing hemarthrosis are rare, angiographic embolization offers a promising, coagulation factor-saving treatment for joint bleeds unresponsive to replacement therapy.

Rodriguez-Merchan et al. suggested that therapeutic arterial embolization should be considered for large hemophilic pseudotumors to reduce their size and minimize bleeding risks during surgery. However, due to its temporary effects, embolization was conducted around 2 weeks before surgery, allowing for mass shrinkage but insufficient time for vessel restoration [37]. 

### 2.4. Treatments

#### 2.4.1. Available Treatment Options Are Shown in Figure 4

Orthopedic surgery in hemophilic patients carries a high risk of complications, including bleeding and infection, necessitating comprehensive preoperative planning and the involvement of multidisciplinary teams to improve outcomes, as highlighted by Badulescu et al. [38]. Early blood factor therapy is vital to prevent complications in these patients. Arthrocentesis of acute hemarthrosis under hemostatic conditions and strict asepsis is a well-tolerated procedure that expedites recovery from acute joint hemorrhage in hemophilia patients. Exercise is an effective hemophilia management strategy with few complications [18]. 

In elbow arthropathy, measured variables included elbow ROM, bicep strength, arm circumference, and elbow pain. Manual therapy improved arm circumference, flexion, and pain [6]. Sandford et al. reported that 67% of patients with hemophilic elbow arthropathy had poor or no adherence to splint use, hindering the benefits of orthotic treatment [39]. 

The conservative treatment of mild osteochondral damage (mild HA) with hematological prophylaxis, painkillers, COX-2 inhibitors, and intra-articular injections of corticosteroids, hyaluronic acid, PRP, and MSCs are effective options [18]. Functional improvements and better quality of life were noted in patients receiving joint lavage with saline, followed by corticosteroid and hyaluronic acid injections [40]. 

Synoviorthesis, involving the destruction of hypertrophic synovium via radiopharmaceutical infiltration, is advised if three months of medical and physiotherapy treatment fail to control synovitis [41]. Three cases of synoviorthesis failing within six months necessitate open surgery synovectomy [42]. Synovectomy is recommended for recurrent hemarthrosis in chronic hemophilic synovitis. Despite usually being painless, chronic synovitis should be suspected in hemophiliacs who have had multiple hemarthroses in recent months. It can be performed via arthroscopy, chemical, or radiosynovectomy techniques [18]. Van Vulpen considers radiosynovectomy the best risk–benefit option for chronic hemophilic synovitis, with chemical synoviocytes as an alternative. Arthroscopic synovectomy is ideal for patients with single joint involvement and subacute or chronic synovitis unresponsive to conservative measures after 3–6 months [43]. In advanced stages of elbow HA, surgery should be considered after conservative treatment. Available procedures include arthroscopic debridement, radial head resection, ulnar nerve transposition, removal of heterotopic ossification, and TEA. Compared to total hip and knee arthroplasties, TEA has inferior outcomes and shorter prosthetic survival rates [18]. 

#### 2.4.2. Hematological Treatment

Hemophilia should be diagnosed promptly by informing pediatricians and parents of the risk associated with family history and initiating treatment in specialized centers immediately. Photodiagnosis of red blood cells, which show lower porphyrin content and enzyme deficiencies in these patients, can facilitate early detection [44]. The primary therapeutic goal is to prevent and treat bleeding in patients with low clotting factor levels. Specific factors can be highly effective as adjuvant therapies during surgical procedures like orthopedic surgeries [45]. 

For patients with HA and frequent bleeding (mostly severe hemophilia), standard care involves regular prophylactic intravenous infusions of factor VIII (FVIII) to maintain FVIII activity levels of ≥1 U/dL, preventing bleeding and long-term complications. However, about 30% of these patients develop neutralizing alloantibodies (FVIII inhibitors), making FVIII replacement therapy ineffective. Before bispecific monoclonal antibody development, patients were treated with prothrombotic coagulation factors that bypassed FVIII, such as activated prothrombin complex concentrate (aPCC) and recombinant activated human factor VII (rFVIIa). These bypassing agents (BPAs), however, have suboptimal hemostatic effects and unfavorable pharmacokinetics, including a short half-life and slow intravenous infusion rate.

Emicizumab (HEMLIBRA^®^; F. Hoffmann-La Roche Ltd., Basel, Switzerland) is a humanized bispecific monoclonal antibody that replicates the cofactor function of absent activated FVIII (FVIIIa), linking activated factor IX (FIXa) and factor X (FX) to restore hemostasis. Lacking sequence homology with FVIII, it is unlikely to induce FVIII inhibitors and remains effective in their presence. Emicizumab offers high subcutaneous bioavailability and a half-life of about 30 days, allowing for once-weekly (1.5 mg/kg), biweekly, or monthly subcutaneous dosing, thus eliminating the need for frequent intravenous administration [46]. 

#### 2.4.3. Open Debridement

**Figure 5:**
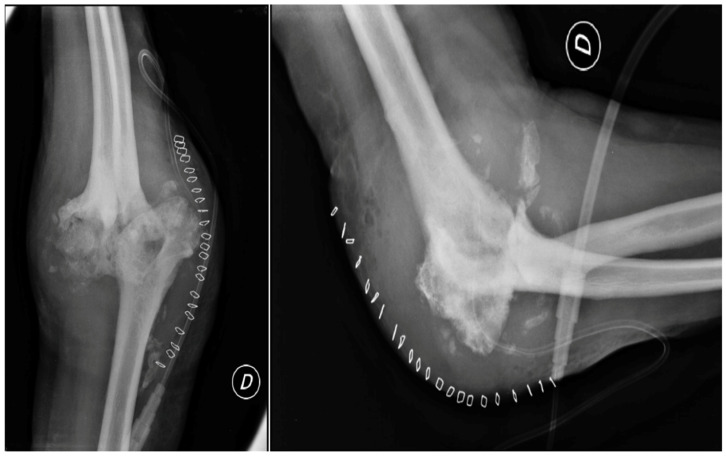
Post-synovectomy and debridement surgery, anteroposterior and lateral views of the right elbow. Reduction in the periarticular mass but persistent joint dislocation.

The patient in question underwent an open debridement with synovectomy and arthroscopy in April 2012 to address the advanced stage of hemophilic elbow arthropathy. During the procedure, the surgeon observed bone resorption with free fragments in the joint, primarily in the radial head, and a thickened capsule with evidence of synovitis. The dislocation was structured and non-reducible. Samples of bone and synovium were collected and cultured for pathogenic microorganisms, which returned positive results for different types of coagulase-negative Staphylococcus. Postoperative radiography revealed the removal of some fragments at the joint level, but the elbow joint dislocation persisted as shown in Figure 5. Synovium fragments showed fibrosis, calcification, scattered foci of chronic inflammation, synovial hyperplasia, and abundant superficial fibrin stores. The bone samples displayed trabecular remodeling and peritrabecular fibrosis with spared perivascular reactive lymphoid aggregates.

Evidence from the Scientific Literature

HA presents with histological evidence of severe joint damage, synovial proliferation, neo-angiogenesis, cartilage and subchondral bone destruction, and osteoporosis, accompanied by intra-articular iron deposition [47]. In hemophilic patients with significant anatomical alterations in bone and soft tissue, intraoperative tissue sampling and cancer biopsies are crucial to exclude other conditions, such as chondrosarcoma, liposarcoma, and synovial sarcoma, which may resemble hemophilia pseudotumors [48]. 

##### Joint Debridement and Complications

After discharge, the patient was instructed to take tranexamic acid every 12 h for 10 days and undergo physiotherapy for active and passive mobilization in flexion, extension, and supination following discharge. The patient was advised to monitor their inflammation index every seven days until it returned to normal levels, and antibiotic therapy was initiated based on the antibiogram. However, the patient was readmitted to the hospital a week later due to a hematoma in the right elbow. Sterile aspiration drainage was performed, and the patient was discharged with an articulated elbow brace set between 10° and 80°, allowing for active mobilization within the range of motion. At subsequent outpatient clinic visits, the wound displayed proximal dehiscence with serum-corpuscular material secretion, and the elbow was flush and swollen, leading to arthrocentesis and the continuation of antibiotic therapy with amoxicillin-clavulanate 1 g per 3/day.

After 15 days, the patient exhibited signs of improvement with no evidence of inflammation; however, there was a persistent wound dehiscence of 0.5 cm and serum corpuscle material discharge. Antibiotic therapy was continued, and the elbow was immobilized in a brace at a 90-degree angle. After 40 days, the wound dehiscence persisted, leading the medical team to discontinue antibiotic therapy and schedule the patient for elbow arthrodesis (a surgical procedure to fuse the bones of a joint), as recommended by the infectious disease specialist. The patient was advised to return for a weekly follow-up to monitor the inflammation index and undergo a CT scan of the elbow. The erythrocyte sedimentation rate (ESR) and C-reactive protein (CRP) levels were 7 in the first hour and 0.35 mg/dL, respectively.

The CT scan, conducted using a 64 md spiral method, revealed significant and widespread osteo-structural abnormalities of a sclerotic nature. This resulted in articulatory deformities that affected all joint bone segments, causing the loss of joint relationships and joint deformities. The scan also revealed multiple periskeletal bone fragments and significant swelling due to soft tissue alterations. The clinical presentation was consistent with advanced HA; however, arthrosinovitis of infectious origin was ruled out.

In late June, due to fluctuating swelling at the back of the elbow, 50 cc of blood fluid was aspirated. Subsequently, in early July, arthrocentesis was performed, which evacuated 100 cc of serum with fibrin deposition. However, there were no signs of elbow infections. The ESR and CRP levels were 12 and 0.56 mg/dL, respectively.

Evidence from the Scientific Literature

Complications of open debridement include wound infection, shoulder–hand syndrome, deep wound infection, ulnar nerve symptoms, radial nerve palsy, residual loose bodies, hematoma, and recurrent effusions [49]. The overall complication incidence is 6.1% (range 0–25%) in the literature, with neurological issues being the most common at 1.9% (range 0–12%). The rate of deep infections is 0.7% (range 0–10%). Most neurological complications respond to neurotrophic drugs or resolve spontaneously, though some require surgical intervention with mixed outcomes. Prevention and targeted antibiotic therapy based on antibiograms are crucial for managing superficial and deep infections [50]. 

#### 2.4.4. Arthrodesis

**Figure 6:**
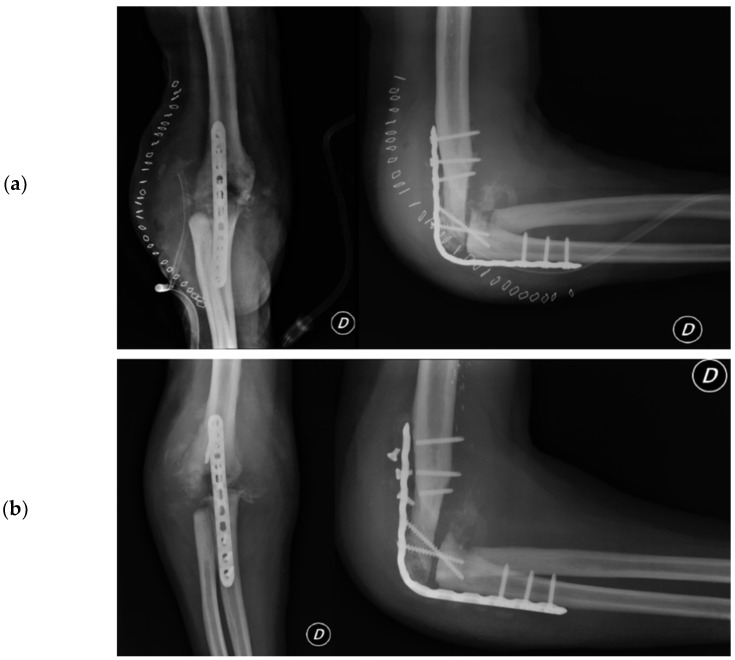
Post-arthrodesis X-ray anteroposterior and lateral views of the right elbow. (**a**) Post operative images showing the plate in site. (**b**) Thirty days after images with mobilization of the synthesis with broken screws.

In August of 2013, a surgical operation was conducted to fuse the elbow joint using a plate with twelve holes and a ninety-degree flexion angle, utilizing the same surgical access point as the previous operation (as depicted in Figure 6). During the course of the procedure, the anatomical structures of the ulna, radial head, and distal humerus were significantly distorted and unidentifiable. New synovectomy and osteotomy were carried out to apply the plate. Following the patient’s release from the hospital, they were instructed to wear a brace at a ninety-degree angle and an arm sling, undergo physiotherapy to move the fingers and wrist, and undergo an X-ray examination thirty days post-surgery.

Evidence from the Scientific Literature

Elbow function loss significantly affects daily activities, occasionally necessitating elbow arthrodesis due to persistent impairment. Fusion rates with current techniques range from 50% to 100%, with increased compensatory motion in the wrist and spinal column. The optimal arthrodesis location remains debated. The primary causes of arthrodesis include post-traumatic arthritis, instability, or infection with joint destruction. No ideal angle exists for elbow fusion, but the literature suggests a range between 45° and 110°, with 90° historically accepted as optimal. Factors influencing the fusion position include sex, occupation, dominant limb, opposite upper extremity functionality, and patient functional requirements. Additionally, ipsilateral shoulder and wrist pathologies and patient preference should be considered. Bracing or casting the elbow at various angles is recommended to determine the optimal fusion position, allowing patient feedback on the best arthrodesis angle [51]. 

##### Arthrodesis and Complications

**Figure 7:**
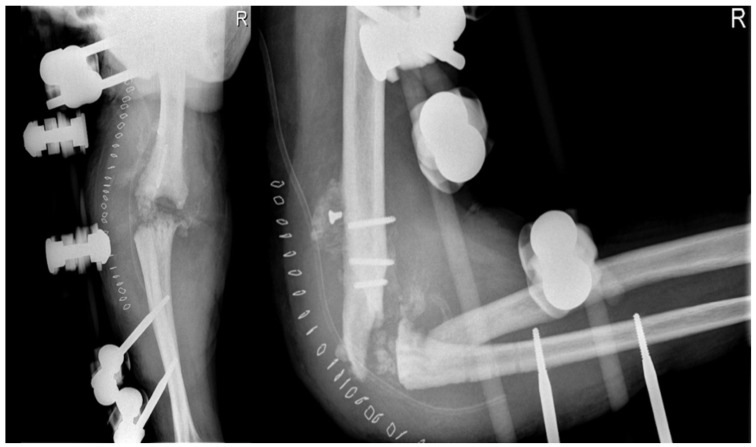
X-ray anteroposterior and lateral views of the right elbow after the External Fixator positioning to stabilize the elbow joint.

At the 30-day postoperative follow-up, radiography revealed three broken screws in the proximal humeral shaft and a subtle periosteal reaction around the plate. The patient was referred for fixation revision and was protected with a new brace. The screws and plates were removed using the previous lateral elbow access. However, internal fixation was not feasible due to the resemblance of the last 7–8 cm of the distal humerus to a cortical lamina. Consequently, an external fixator, bar-to-bar, was applied after the arthrodesis surfaces were prepared. as shown in Figure 7, the follow-up radiograph confirmed the appropriate positioning of the device. At hospital discharge, the orthopedic recommendation was to wear the external fixator for 60 days, rest, and undergo weekly ambulatory assessments of local clinical findings.

Evidence from the Scientific Literature

Elbow external fixators can be used for temporary stabilization or as a hinged motion fixator. Common surgical indications include temporary stabilization in cases of damage control surgery, such as fractures with extensive soft tissue damage or multiple trauma patients, additional protection/motion control after complex osteoligamentous surgery, or isolated applications in cases of concomitant injuries or comorbidities, persistent intraoperative dislocation tendency after osteoligamentous stabilization, and distraction treatment/distraction arthrolysis. An accurate center of rotation is crucial, as it affects the range of motion, adding complexity to the procedure. Pins can cause soft tissue irritation, loosening, malpositioning, fracture/pull, infection, and hypertrophic scarring. Arterial and nerve damage, particularly to the radial nerve, is a common surgical risk, necessitating a thorough understanding of neurovascular anatomy. Safe soft tissue preparation with proper exposure of the humerus and ulna is preferred over risky stab incisions. Postoperative complications include periarticular calcification/heterotopic ossification, secondary loss of reduction, persistent instability, osteitis/osteomyelitis, residual pain, limited movement, and specific complications of the underlying pathology [52]. 

#### 2.4.5. Total Elbow Arthroplasty

**Figure 8:**
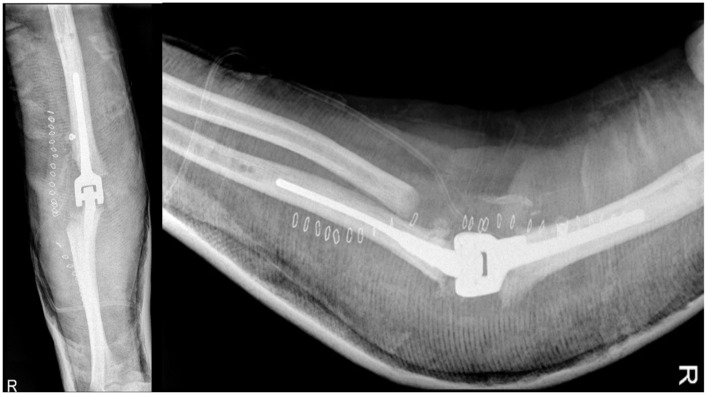
X-ray anteroposterior and lateral views of the right elbow after the placement of the total elbow arthroplasty (TEA) with the restoration of normal joint relationships.

The patient utilized the external fixator for a period of two months in order to facilitate the healing of their soft tissue. Following this period, the patient underwent TEA surgery in November of 2013. During the procedure, the broken screws were removed and the distal epiphysis of the humerus and ulna were prepared for the implantation of a Coonrad–Morrey prosthesis with a humeral stem extra small/L 100 mm cemented. The extensor apparatus of the ulna was stabilized with a non-resorbable fiber wire, and post-operative X-rays revealed the proper placement of the prosthesis (shown in Figure 8). The patient was instructed to maintain a 90-degree cast on their elbow until the wound had healed, followed by careful mobilization. Tranexamic acid was prescribed for a week, and the wound-healing process was successful, with the patient achieving a range of motion in flexion of approximately 100 degrees fifteen days after the TEA surgery.

Evidence from the Scientific Literature

When arthrodesis failed, prosthetics were reconsidered. Burkhart et al. [53] effectively described the transition from arthrodesis to prosthesis. The first elbow prosthesis was developed by R. Dee in 1972, and Coonrad later introduced a bone-sparing version. Morrey modified this prosthesis in 1978, incorporating a central pivot joint instead of the native C-lock [54,55]. 

The primary indication for total elbow arthroplasty (TEA) is elbow arthritis, often due to rheumatoid arthritis, in patients over 65 with limited functional demands and severe pain, significant loss of range of motion (ROM), impaired elbow function, and failed nonoperative treatments. Relative indications include arthrosis, post-traumatic arthrosis, and comminuted fractures in elderly patients where internal fixation is not feasible [56]. Other indications include large bone loss, HA [56], and reconstruction following tumor resection.

Contraindications include active infections, neuromuscular paralysis, and open wounds. Relative contraindications are non-compliant patients, those engaged in heavy work, massive bone loss, and functional, non-painful arthrodesis.

Complications involve infections (2–4%) and ulnar neuropathy (5%), often requiring transposition due to extensor apparatus insufficiency, treatable with an anconeus flap or Achilles tendon allograft. Instability, with a 15% rate of dislocation or subluxation, is managed with splinting if occurring within weeks post-surgery or revision with a linked prosthesis if later. Mechanical failures include a 2% rate of aseptic mobilization in Coonrad Morrey prostheses, 8% in Souter, and 18% in Kudo.

The elbow prosthesis can be linked (with a central pin joint system) or nonlinked. Linked prostheses allow for extensive soft tissue release, enhance the range of motion, and provide greater joint stability despite significant bone loss and ligament insufficiency. However, they increase tension on joint surfaces, potentially leading to prosthesis loosening (the detachment or instability of an implanted joint replacement). After initial immobilization in extension, early unprotected mobilization is allowed with a linked prosthesis, depending on the wound extent and extensor apparatus reconstruction. If significant flexion contracture is detected pre-surgery, an extension splint should be worn at night for several weeks.

A Mayo Clinic review of prostheses for rheumatoid arthritis reported that out of 461 implants over 2–25 years, 43 required revision: 10 for infection, 25 for mobilization, 8 for polyethylene degeneration, and 3 for periprosthetic fractures. For post-traumatic prostheses, 16 out of 85 failed. Prostheses are recommended for pseudoarthrosis of the distal humerus, particularly in elderly patients with limited bone stock. The Mayo Clinic study on 92 implants for distal humerus pseudoarthrosis with 2–20 years of follow-up revealed that 79% of patients were pain-free with a ROM of 22° to 135°. Sixteen patients experienced aseptic mobilization, five had prosthesis ruptures, and five had deep infections [57]. 

In a study of elbow arthroplasty for HA, involving seven patients with an average age of 34, three were treated with a Coonrad Morrey prosthesis, and only one required revision for pain and clicking, lasting 12 years post-revision. This retrospective study had an average duration of 118 months, with other patients showing improvements in pain and ROM [58]. 

TEA in patients with advanced HA is linked to higher complication and revision rates compared to patients without bleeding disorders, but it provides good functional and subjective long-term outcomes. Indications for TEA have expanded with increased knowledge and have become favorable for patients with advanced HA. However, reports on long-term outcomes of TEA in HA remain limited [59]. 

##### Total Elbow Arthroplasty and Complications

**Figure 9:**
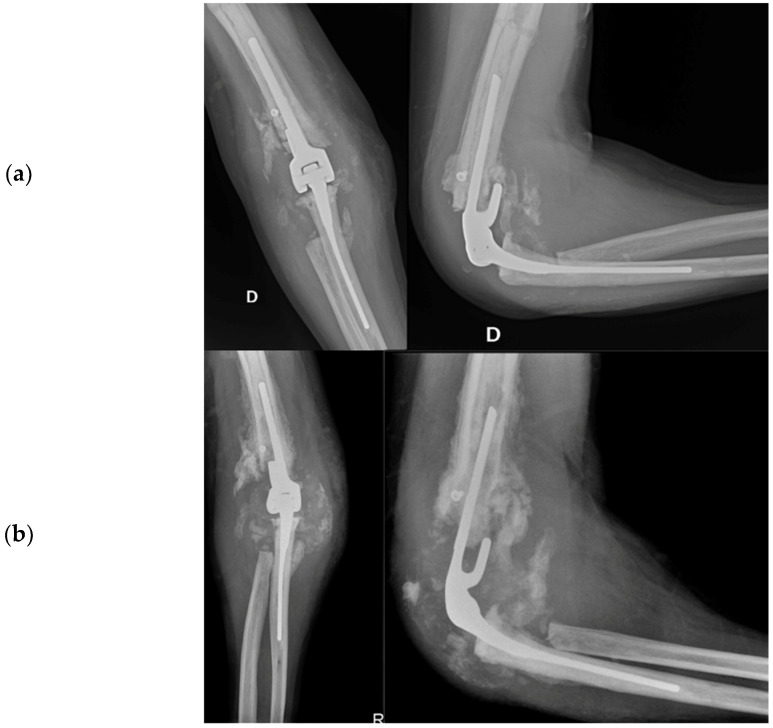
(**a**) Radiographic X-ray anteroposterior and lateral views: beginning of prosthesis loosening with increased bone radiolucency around the prosthesis; (**b**) 30 days after loosening of the prosthesis and increasing of periarticular calcifications.

Two months post-TEA surgery in February 2014, the patient faced complications despite satisfactory elbow flexion (approximately 120°). Calcific deposits increased, and a faint radiolucent line appeared around the prosthetic humeral stem, with no changes to the ulnar stem. A radiograph taken 15 days later showed humeral TEA loosening, increased radiolucency at the apex and along the stem, caudal dislocation, and resorbed calcific deposits as shown in Figure 9a. The patient was also hospitalized for Hodgkin’s lymphoma recurrence, presenting with local swelling and elevated inflammatory markers: ESR at 41, CRP at 7.68 mg/dL, and WBC at 10.06 thousand/mm^3^.

A subsequent CT scan confirmed humeral stem loosening, caudal displacement, and mediolateral tilting. Significant bone loss around the prosthesis was evident, with lytic bone remodeling and periosteal reaction. The ulnar portion was well fixed but showed periosteal reaction. Intra-articular loose bodies, capsular calcifications, and soft tissue swelling were also observed. The radiologist suspected advanced HA but could not exclude arthritic synovitis of infectious origin or lymphoproliferative disease.

After the patient’s elbow became swollen and hot with limited ROM and an audible click, an open elbow cast was applied, a blood culture was taken, and empiric antibiotic therapy with Trimethoprim/sulfamethoxazole (160 mg/800 mg) and Minocycline (100 mg) was initiated. However, a follow-up radiograph after 15 days showed an increase in joint calcifications and no improvement in implant loosening, as shown in Figure 9b.

Evidence from the Scientific Literature

Postoperative complications of TEA include infection, ulnar nerve neuropathy, compartment syndrome, polyethylene wear, periprosthetic infection, and loosening of the humeral component. The long-term functional benefits, such as enhanced range of motion and patient-reported outcomes, indicate that semi-constrained linked TEA is effective for treating HA of the elbow in a highly selected population, though a complication rate of 62% and revision rate of 38% should be expected [59]. 

Despite the growing use of TEA, long-term issues like infection, aseptic loosening, instability, and periprosthetic fractures remain problematic. This design allows some varus–valgus motion, reducing stress at the bone–cement interface, and has been used effectively in conditions such as rheumatoid arthritis, degenerative arthritis, and trauma reconstruction, with satisfactory long-term outcomes. However, aseptic loosening due to bushing wear is a major cause of implant failure, and lowering the complication rate remains challenging. Revision-related problems have increased alongside primary elbow arthroplasty, and it is well established that patients with inflammatory arthritis have longer-lasting TEA outcomes compared to those with trauma-related causes. Patients with significant comorbidities, smoking, obesity, and young age have an elevated risk of complications. In constrained arthroprostheses, multiple revisions are due to polyethylene wear, whereas in unconstrained prostheses, revisions are often due to instability and dislocations. Infection, aseptic loosening, and periprosthetic fractures are the primary complications necessitating revision surgery. Aseptic loosening is a common cause of revision, frequently resulting from stress shielding-induced osteolysis around the implant. According to Wolff’s law, nonanatomic force transmission in TEA leads to stress shielding at the humeral condyles and olecranon, causing bone resorption and increasing the moment of force on the arm. This predisposes patients to stem loosening and arthroplasty failure due to polyethylene wear, mechanical failure, or periprosthetic fracture. Revision TEA for loose stems is feasible with larger stems, and bone grafting can be performed if needed. King reported on 31 patients who underwent revision TEA with a semi-constrained prosthesis for aseptic loosening, with a mean 6-year follow-up, achieving a mean MEPS of 87 and a mean flexion-extension arc of over 100° [60]. 

A preliminary diagnosis relies on clinical examination, basic imaging, and serological tests. Presentations fall into three categories: TEA may be stiff (reduced active range of motion), weak (reduced active power of motion), or unstable (prosthetic loosening or dissociation). These conditions may coexist with or without infection. Diagnosing stiffness due to infection, implant impingement, malrotation, or heterotopic ossification requires anesthesia. Fluoroscopy and joint aspiration are valuable diagnostic tools. Nerve conduction tests and ulnar nerve ultrasonography (US) with Doppler augmentation identify causes of intrinsic neuropathy and extrinsic nerve compression. Bone length loss, subduction of a loose implant, and imaging of the triceps mechanism help evaluate if the TEA is weak and deteriorating. Stress radiographs assess a painful, unstable TEA due to a failing linking mechanism or insufficient collateral ligaments in an unlinked implant. Computed tomography (CT) is crucial for assessing bone loss and implant alignment. In all cases, infections must be considered and investigated.

Patients with fever, erythema, wound dehiscence/blister with sinus or fistula formation, persistent or worsening local discomfort, swelling, or radiolucent lines on radiographs of the bone–cement–implant interface may have a periprosthetic elbow joint infection. Identifying infectious microorganisms is crucial for guiding antimicrobial treatment and implant management. US investigation with guided aspiration and CT imaging can help detect infections in the extracapsular compartment (skin and subcutaneous tissues, including the triceps), intracapsular compartment, or intraosseous compartment (osteomyelitis). Key considerations for planning revision surgery include the condition of the neurovascular system, soft tissues (skin and muscle–tendon envelope), bone quality after implant removal, implant stability in the bone, and presence or absence of infection [61]. 

#### 2.4.6. Revision Total Elbow Arthroplasty

In January 2014, a surgical procedure was conducted via the conventional lateral access approach to replace the prosthesis with a Coonrad–Morrey humeral stem small/L 150 mm/Flangia by thorough irrigation and cementation in the absence of any signs of infection in the operating room. The patient was instructed to keep the arm in a sling for a month. Due to the arthrodesis failure and the good fit of the ulnar component, prosthetic revision was deemed the most appropriate option. Postoperative radiography demonstrated appropriate implant placement.

**Figure 10:**
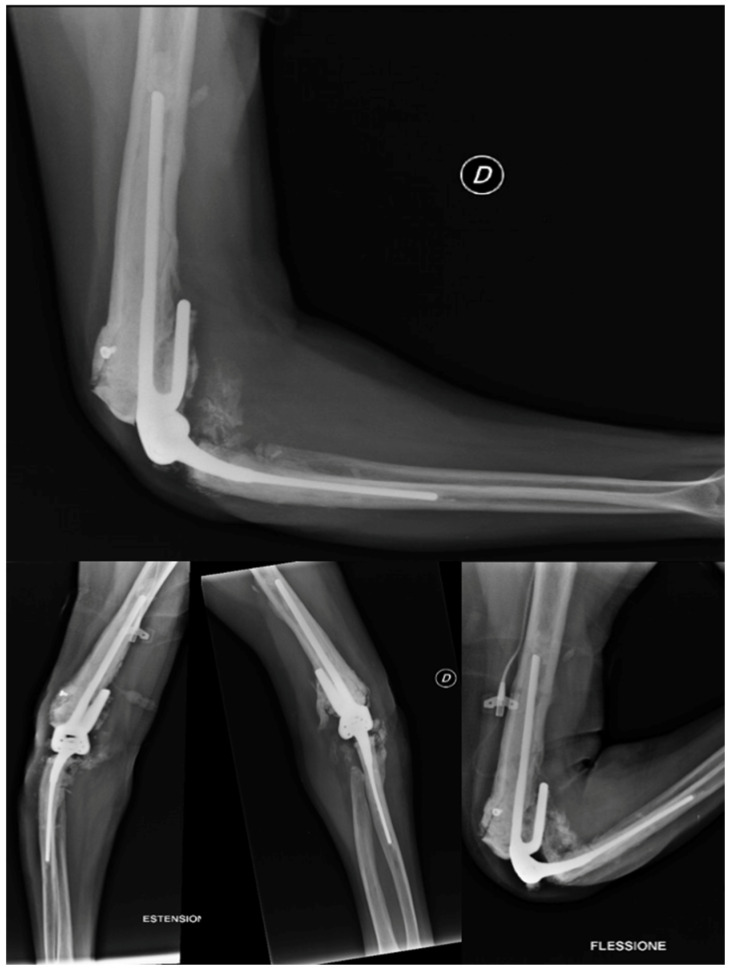
X-ray anteroposterior, lateral in neutral position, in extension and in flexion views of the right elbow at 6 months after the second revision surgery. No signs of loosening. Good alignment of the implant in neutral position and in flex–extension position.

The patient had a good postoperative course, except for a small wound dehiscence with fibrinous serum secretions, which resolved in May 2014 with outpatient clinic treatments. In July 2014, after four months, the patient achieved complete extension and flexion of 100°, with no significant changes in the radiographic findings. Two months later, in September 2014, the radiographs remained unchanged, as shown in Figure 10, and the patient had a flexion of approximately 105°, complete extension and pronation, and limited supination of 5°.

Evidence from the Scientific Literature

Postoperative follow-up in complex revision surgery relies on the reconstructed extensor mechanism’s condition and the initial stability of the bone–implant structure. Skin wound healing, reduction in distal limb edema, hand mobility, and ulnar nerve protection are crucial for managing the postoperative recovery of flexion–extension and pronation–supination. A circumferential bandage must be used carefully to avoid distal pain and edema. Isometric activation of shunt muscles (biceps and triceps) enhances joint stability and promotes neural feedback mechanisms of motor control. Functional rehabilitation spans from 3 weeks to 3 months, from wound healing to extensor system recovery. The principle that the shoulder and elbow are “servants of the hand” guides rehabilitation: the elbow and shoulder follow hand tasks. Weight-bearing and load-sharing in long lever arm activities can begin once implant-bone stability is achieved, typically no earlier than 3 months post-surgery, depending on patient needs and goals. High forces and impacts should generally be avoided. Biomechanical studies have not substantiated recommendations to avoid compression or distraction loads exceeding 2 kg. The literature indicates that up to 40% of patients with elbow replacement engage in activities “excessively demanding” for TEA, despite relevant advice. Given the multifactorial nature of TEA failure, revision total elbow arthroplasty (R-TEA) remains a significant challenge, necessitating careful evaluation of the patient, their environment, functional goals, and an in-depth understanding of elbow biomechanics [61]. 

## 3. Discussion

**Figure 11:**
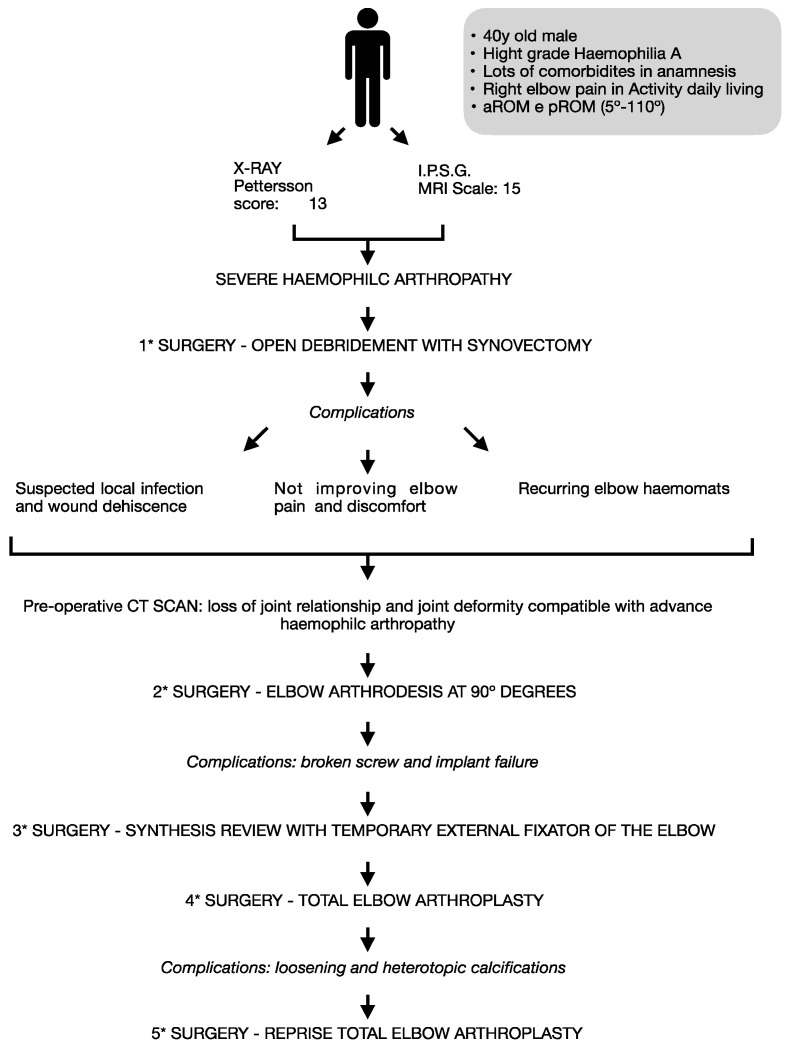
Case report flow chart.

This case report provides a valuable example of clinical practice by illustrating multiple surgical and treatment interventions for a single patient, accompanied by associated complications, as schematized in Figure 11. The patient, a 40-year-old male with severe hemophilia A, presented with significant elbow arthropathy compounded by comorbidities such as lymphoma and smoking. Due to the patient’s high functional demand and severe joint instability, conservative management was deemed unlikely to produce favorable outcomes. In such cases, conservative approaches are associated with a high risk of recurrent hemarthrosis and rapid progression of joint destruction, as supported by multiple studies on hemophilic arthropathy [62,63]. 

With the failure of conservative measures, surgical intervention was pursued to address both the joint instability and the advanced degenerative changes. It was challenging to perform arthroscopic synovectomy in this patient due to the anatomic joint alterations caused by HA, although this procedure has been described and used in the treatment of this condition [63]. In cases of advanced arthritis where arthroscopy is technically challenging, open synovectomy and debridement of calcifications present a viable alternative [64,65]. Based on our experience, this approach is advisable in patients with severely compromised anatomy.

The occurrence of postoperative hematoma may have been exacerbated by the patient’s underlying coagulopathy and was managed with aspiration and continued antibiotic therapy, though wound dehiscence persisted, indicating the complexity of surgical management in hemophilic patients. In this case, an open synovectomy was conducted to remove part of the atypical soft tissue around the joint. Preoperative artery embolization, ideally two weeks before surgery, would have helped control intraoperative bleeding and prevent postoperative complications [48].

The postoperative hematoma was managed through aspiration and continuous factor replacement therapy, following the guidelines for surgical management in hemophilia [66]. Despite these efforts, wound healing was prolonged, likely due to the patient’s compromised immune status secondary to lymphoma. Orthopedic surgery in hemophilic patients presents unique challenges due to the increased risk of bleeding, infection, and poor wound healing, as highlighted by Badulescu et al. [38]. The systematic review emphasizes the importance of comprehensive preoperative planning, multidisciplinary care, and postoperative monitoring to mitigate these risks, particularly in complex cases such as hemophilic elbow arthropathy.

The decision to proceed with arthrodesis was influenced by the patient’s young age, high functional demand, and persistent joint instability, despite previous interventions. Arthrodesis has been shown to provide long-term stability and pain relief in patients with advanced joint destruction [67]. Revision TEA was considered as an alternative, but it was ultimately deemed less advantageous for the patient due to several factors: severe soft tissue compromise, significant joint deformity, and the increased invasiveness of the procedure, which would have heightened the risks of complications such as bleeding, neurovascular injury, and infection. Compared to arthrodesis, revision TEA posed a greater surgical challenge, particularly in this complex patient. Furthermore, the patient’s functional needs prioritized pain relief and joint stability over mobility. For these reasons, arthrodesis was chosen, though perhaps the decision was overly influenced by concerns about known complications, leading to an underestimation of the difficulties associated with achieving fusion in such a complex case.

Arthrodesis failure was likely due to factors such as a too-short humeral plate, lack of bone grafts, insufficient locking screw length, and early arm mobilization. An external fixator was used for surgical wound dehiscence, and total elbow arthroplasty was reconsidered after arthrodesis failure.

The progression from arthrodesis to TEA was largely influenced by the failure of the arthrodesis to provide sufficient stability and pain relief, despite initially being selected due to the patient’s young age and high functional demands. Persistent complications, such as poor osteointegration (the process by which the bone grows and integrates with the surface of a prosthesis), humeral plate failure, and ongoing pain, necessitated the transition to TEA, which was expected to provide better stability and functional outcomes. However, the presence of severe soft tissue compromise and extensive bone loss made the TEA procedure technically challenging and carried a heightened risk of early prosthesis loosening, which unfortunately occurred in this case.

Burkhart et al. [53] described the transition from arthrodesis to prosthesis as successful. This study utilized the Coonrad–Morrey model of linked TEA for its enhanced joint stability, even in cases of significant bone loss and ligament insufficiency, while maintaining a good range of motion. However, increased joint surface tension may cause early prosthesis loosening. After surgery, the patient was instructed to keep the wound dry and to gradually begin cautious elbow mobilization. Additionally, the patient was prescribed tranexamic acid, two oral vials every 12 h for one week. Initial immobilization in extension for a few days, followed by cautious mobilization, was recommended. Prosthesis loosening was observed, likely due to the undersizing of the humeral stem, combined with poor osteointegration potentially exacerbated by local inflammation and the patient’s comorbid conditions. 

The challenge of prosthesis sizing, particularly in cases of significant bone loss, highlights the need for meticulous preoperative planning and imaging to avoid undersizing, which likely contributed to the prosthesis mobilization encountered postoperatively.

The decision to proceed with revision surgery following the failure of the TEA was driven by the continued prosthesis loosening and the patient’s deteriorating functional status. Despite the increased risks associated with revision surgery in hemophilic patients, it was deemed necessary to attempt to restore joint stability and function. This case underscores the complexity of managing hemophilic elbow arthropathy and the need for adaptive surgical strategies when initial interventions fail to achieve their intended outcomes.

Given the failure of the arthrodesis and the incorrect positioning of the ulnar component, which led to aseptic loosening, prosthetic revision was the most reasonable option. The patient’s extensive bone destruction in the distal humerus upon admission only worsened after subsequent surgeries, suggesting that a more radical surgical approach, such as direct elbow prosthesis implantation, might have been the better choice from the outset. This option has shown good results in the literature, even in younger patients [58,68,69].

This case highlights the complexity of surgical management in hemophilic patients with advanced joint disease and multiple comorbidities. Comprehensive preoperative planning, including detailed imaging, computed tomography–based three-dimensional preoperative planning [36], and multidisciplinary input are essential in minimizing the risk of complications and optimize surgical outcomes. The importance of early surgical intervention, tailored to the patient’s specific needs and lifestyle, cannot be overstated. Future research should focus on refining surgical techniques and preoperative protocols to further improve outcomes in this challenging patient population.

From a psychological perspective, the patient experienced understandable frustration and anxiety following multiple surgeries and complications, including the failure of arthrodesis and subsequent prosthesis loosening. The uncertainty of the outcomes and the prolonged recovery led to significant emotional distress. However, with continued support from the multidisciplinary team, including psychological counseling and reassurance, the patient demonstrated remarkable resilience. Although the emotional burden was heavy, the patient remained determined to pursue a positive outcome and was ultimately grateful for the care received. The inclusion of mental health support during complex, prolonged treatment processes proved crucial in addressing the emotional challenges alongside the physical recovery.

## 4. Conclusions

The management of hemophilic patients with elbow arthropathy requires a multidisciplinary approach to prevent and manage complications effectively. In cases with significant soft tissue and bone compromise, joint replacement should be strongly considered as the first-line treatment, despite its higher complication rates, as other surgical methods often yield variable outcomes with similar risks. Preoperative measures, such as embolization for pseudotumors, should be implemented when necessary to reduce complications.

Future management should focus on leveraging advanced preoperative planning technologies to avoid technical issues like undersizing of prosthetic components and to minimize surgical time. These strategies will be key to improving outcomes for hemophilic patients with complex joint disease.

## Figures and Tables

**Figure 4:**
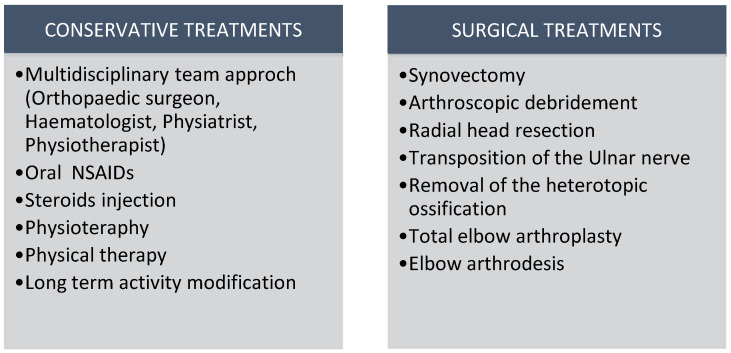
Available treatment options for HA. (NSAIDs: nonsteroidal anti-inflammatory drugs).

## Data Availability

The original contributions presented in the study are included in the article, further inquiries can be directed to the corresponding author.
